# Primary Central Nervous System Lymphoma With Testicular and Prostatic Involvement: A Case Report

**DOI:** 10.7759/cureus.101783

**Published:** 2026-01-18

**Authors:** Javier A Rodríguez Morales, Valeria Murguía Salgado, Valdemar Barajas López, Alejandra Alvarez Salinas, Lauro F Amador

**Affiliations:** 1 Department of Medicine and Nutrition, Universidad de Guanajuato, León, MEX; 2 Department of Internal Medicine, Hospital General de San Juan del Río, Querétaro, MEX; 3 Department of Anesthesiology, Universidad Nacional Autonoma de Mexico, León, MEX; 4 Department of Hematology, Hospital Regional de Alta Especialidad del Bajío, Servicios de Salud del Instituto Mexicano del Seguro Social para el Bienestar (IMSS-Bienestar), León, MEX; 5 Department of Research, Unidad Médica de Alta Especialidad Hospital de Especialidades No. 1 Centro Médico Nacional (CMN) Bajío, León, MEX

**Keywords:** central nervous system tumor, diffuse large b-cell lymphoma (dlbcl), non-hodgkin lymphoma, prostate tumor, testis cancer

## Abstract

Diffuse large B-cell lymphoma (DLBCL) is an aggressive subtype of non-Hodgkin lymphoma that most commonly presents with nodal disease, while primary involvement of the central nervous system (CNS) is rare, particularly in immunocompetent patients. We describe the case of a 41-year-old immunocompetent male who presented with progressive left-sided hemiparesis, ipsilateral facial palsy, severe headache, and decreased level of consciousness. Neuroimaging findings and histopathological examination confirmed the diagnosis of primary CNS DLBCL. Subsequent systemic evaluation with PET-CT revealed increased metabolic activity in the testicular and prostatic regions, consistent with secondary extranodal involvement. The patient was treated with a MATRIX-based chemotherapy regimen, including high-dose methotrexate, cytarabine, and rituximab, in accordance with current guidelines for primary CNS lymphoma. This case highlights an unusual presentation of primary CNS DLBCL with early systemic dissemination to testicular and prostatic tissue, underscoring the importance of comprehensive staging and prompt initiation of high-dose methotrexate-based therapy to optimize clinical outcomes.

## Introduction

Primary central nervous system lymphoma (PCNSL) is a rare type of extranodal non-Hodgkin lymphoma that exclusively affects the brain, spinal cord, leptomeninges, or vitreoretinal space, with no evidence of systemic disease at diagnosis [1-5]. Its annual incidence is approximately 0.4 per 100,000 inhabitants, increasing to one per 100,000 in people over 70 years of age, with a median age at diagnosis between 56 and 61 years. Most cases correspond to the histological subtype of diffuse large B-cell lymphoma (DLBCL) [1,4]. The pathogenesis involves recurrent genetic alterations affecting B-cell receptor, toll-like receptor, and NF-κB signaling pathways, with frequent mutations in MYD88 and CD79B, and deletions in CDKN2A and HLA-D loci contributing to immune evasion and tumorigenesis [1,6,7].

Diagnosis requires high clinical suspicion, as manifestations depend on tumor location. The most common symptoms are focal neurological deficits (50-70%), cognitive or behavioral changes (40-50%), and headache due to intracranial hypertension (33%) [1-3]. Histopathological study and immunohistochemistry are essential to confirm the neoplasm, with expression of pan-B-cell markers including CD19, CD20, PAX5, BCL6, MUM1/IRF4, and CD10 [4,7].

First-line treatment is based on high-dose methotrexate chemotherapy (≥3.5 g/m²), which adequately penetrates the blood-brain barrier, and is commonly combined with cytarabine, rituximab, and thiotepa (MATRIX or R-MVP regimens) [1-4,7]. In our institution, a modified MATRIX regimen is used, omitting thiotepa due to lack of availability. Although radiotherapy, particularly whole-brain radiation therapy, achieves high overall response rates (~90%), these responses are not durable (overall survival 12-18 months) and are associated with relapse and significant long-term neurotoxicity, especially at higher doses [2]. Despite the high chemosensitivity of the disease, 15-25% of patients are refractory to chemotherapy, and relapse occurs in 25-50% of patients after an initial response, with an overall five-year survival of 30-40% [1,2,7].

Extraneural dissemination in PCNSL is uncommon, reported in approximately 4-7% of cases, and carries important clinical implications, as it often reflects advanced disease or relapse. Involvement of immune-privileged sanctuary sites such as the testis or prostate is exceptionally rare and poses diagnostic and therapeutic challenges [1,2]. This case highlights the aggressive clinical behavior of the disease and reinforces the need for early, comprehensive systemic evaluation to optimize therapeutic planning.

## Case presentation

**Figure 1:**
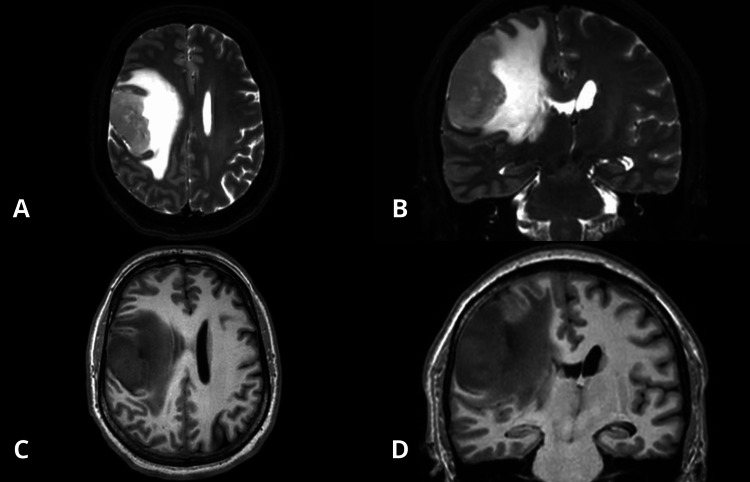
Contrast-enhanced T2 and T1 MRI scan of the skull A: contrast-enhanced T2 axial MRI scan of the skull showing a right frontoparietal lesion with a neoplastic appearance; B: contrast-enhanced T2 coronal MRI scan of the skull showing a right frontoparietal lesion with a neoplastic appearance; C: contrast-enhanced T1 axial MRI scan of the skull showing a right frontoparietal lesion with a neoplastic appearance; D: contrast-enhanced T1 coronal MRI scan of the skull showing a right frontoparietal lesion with a neoplastic appearance.

We report the case of a 41-year-old male with no significant past medical history. In August 2025, he presented with progressive left-sided hemiparesis, ipsilateral facial paralysis, severe headache, and decreased level of consciousness. Brain magnetic resonance imaging (MRI) revealed a right parietal lesion (Figure 1). The craniotomy was performed shortly after radiological diagnosis due to rapid neurological deterioration, and the patient received intravenous corticosteroids prior to surgery to reduce tumor-related vasogenic edema. He subsequently underwent complete resection of the right temporoparietal tumor, achieving gross total resection with an adequate postoperative course.

**Figure 2:**
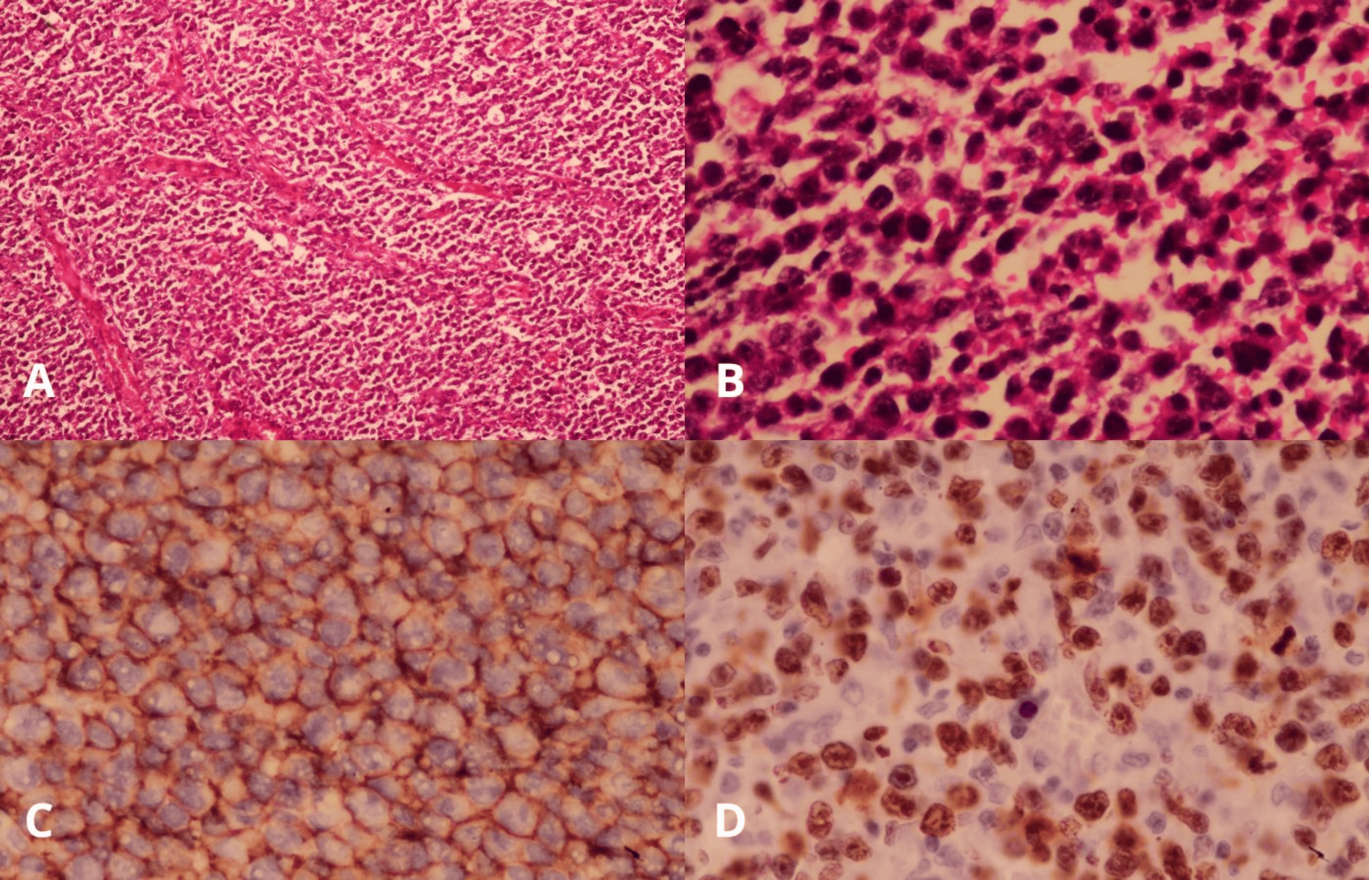
Histopathological and immunohistochemical findings A: low-power hematoxylin and eosin (H&E, ×10) section showing a diffuse, densely cellular lymphoid infiltrate with effacement of the normal tissue architecture; B: high-power view (H&E, ×40) demonstrating sheets of atypical lymphoid cells with scant cytoplasm, enlarged hyperchromatic nuclei, and increased mitotic activity; C: immunohistochemical staining for CD20 showing strong and diffuse membranous positivity in the neoplastic cells, confirming B-cell lineage; D: Ki-67 immunostaining demonstrating a high proliferative index, with nuclear positivity in a large proportion of tumor cells.

Histopathological examination confirmed diffuse large B-cell non-Hodgkin lymphoma, positive for CD20, BCL6, and VL2, with a Ki-67 proliferation index of 90%, findings consistent with primary high-grade CNS lymphoma (Figure 2).

**Figure 3:**
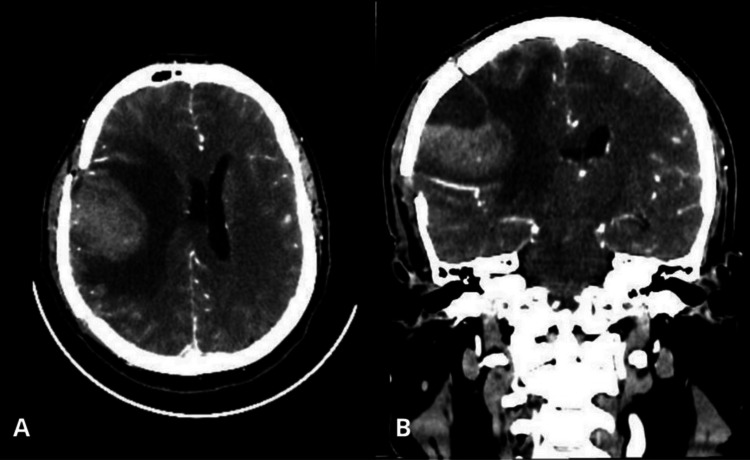
Contrast-enhanced computed tomography in the arterial phase of the skull A: contrast-enhanced computed tomography in the arterial phase of the axial skull showing the cerebral parenchyma with nodular solid imaging at the lobe level; B: contrast-enhanced computed tomography in the arterial phase of the coronal skull showing the cerebral parenchyma with nodular solid imaging at the lobe level.

While the diagnostic evaluation and treatment planning were still ongoing, the patient was readmitted in October 2025 due to acute neurological deterioration, characterized by decreased level of consciousness, oral intolerance, motor deficits, and projectile vomiting. On examination, his Glasgow Coma Scale score was 11/15 (E1, V5, M5); right facial paralysis, right eye deviation, anisocoria, and left hemiparesis were noted. He was hemodynamically stable, with no clinical signs of intracranial hypertension. Follow-up cranial CT demonstrated recurrence of the parietal lesion (Figure 3).

During hospitalization, neurocritical management was guided by GHOST-CAP goals [8], including maintaining blood glucose between (80-180 mg/dL), hemoglobin (7-9 g/dL), oxygen saturation (92-96%), serum sodium (135-155 mEq/L), temperature below 38°C, adequate pain and anxiety control, mean arterial pressure (<80 mmHg), and arterial pCO₂ between (35-45 mmHg). The patient developed a polyuric syndrome, which resolved with desmopressin administration. He remained under close observation for risk of tumor lysis syndrome until completion of further imaging studies.

**Figure 4:**
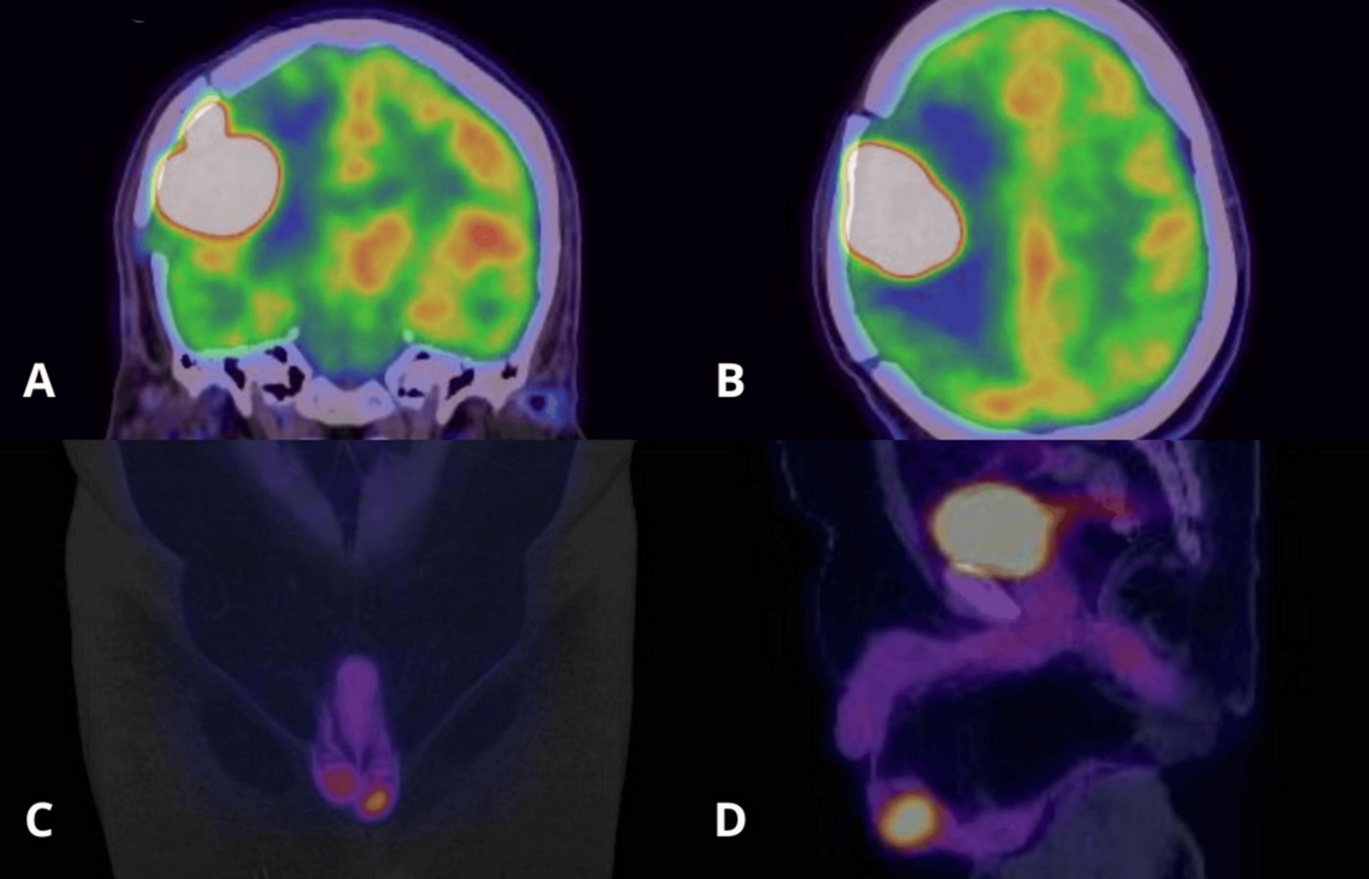
PET/CT A: coronal brain PET/CT post-craniotomy showing a hypermetabolic lesion located in the right frontoparietal region; B: axial brain PET/CT post-craniotomy showing a hypermetabolic lesion located in the right frontoparietal region; C: coronal abdominopelvic PET/CT showing hypermetabolic lesions located in the right and left testes; D: sagittal abdominopelvic PET/CT showing hypermetabolic lesions located in the right and left testes and the prostate.

A PET-CT scan performed on October 22, 2025, revealed a rounded hyperdense lesion in the right frontoparietal cortico-subcortical region with blurring of adjacent sulci and perilesional vasogenic edema, measuring 37 × 38 mm. The lesion caused partial collapse of the ipsilateral lateral ventricle without midline shift and showed intense hypermetabolism (SUVmax 45.28). Additionally, increased ^18^F-fluorodeoxyglucose (FDG) uptake was observed in the left peripheral prostate region (SUVmax 4.60) and in the left testis, which was slightly enlarged compared with the contralateral side and exhibited focal hypermetabolism (SUVmax 11.22), with no associated hydrocele (Figure 4).

Based on these findings, we planned to initiate a MATRIX chemotherapy regimen consisting of methotrexate, cytarabine, rituximab, and thiotepa. However, thiotepa, which is part of the standard MATRIX protocol, was omitted due to its unavailability at our institution. The patient initially showed a good clinical response to treatment; nevertheless, he subsequently died due to infectious complications associated with the second cycle of the MATRIX regimen.

## Discussion

PCNSL is a rare and aggressive extranodal subtype of DLBCL, most commonly affecting middle-aged to elderly adults. Presentation at 41 years of age, as in the present case, is therefore atypical and may have implications for both prognosis and therapeutic response [1]. The tumor’s immunophenotype, CD20 and BCL6 positivity, with a markedly elevated Ki-67 proliferation index of approximately 90%, reflects an aggressive biological behavior associated with rapid tumor growth and poorer outcomes [9].

Neuroimaging remains essential for the initial evaluation of PCNSL. MRI is the cornerstone for lesion characterization and localization, while whole-body FDG PET-CT has emerged as an invaluable tool for staging. PET-CT can reveal occult systemic disease in a subset of patients initially presumed to have isolated CNS involvement, thereby altering disease classification and guiding management [10].

In this case, FDG-avid lesions identified in the testicular and prostatic regions raised significant concern for systemic dissemination to immune-privileged sites. Testicular involvement, in particular, has been associated with a high risk of CNS relapse and warrants targeted evaluation through testicular ultrasound or biopsy to confirm secondary disease [11]. The management plan for this patient with testicular cancer consisted of a radical orchiectomy of the affected testis, along with prophylactic radiotherapy to the contralateral testis at a dose of 25-30 Gy. This approach is associated with improved local control and a reduced risk of contralateral recurrence [12]. However, the planned testicular management could not be carried out because the patient passed away.

Despite initial gross total resection, this patient experienced early postoperative recurrence accompanied by systemic disease progression, both of which are poor prognostic indicators [13]. Established risk models such as the International Extranodal Lymphoma Study Group (IELSG) score highlight age, performance status, deep brain involvement, and extracerebral spread as the principal determinants of survival [10].

## Conclusions

This clinical case describes a patient with primary DLBCL of the CNS, an uncommon and highly aggressive malignancy characterized by atypical dissemination patterns, including early involvement of immune-privileged sanctuary sites such as the testis and prostate. Comprehensive staging with PET-CT proved essential for detecting extracerebral disease and guiding therapeutic decisions. The use of a MATRIX regimen remains a cornerstone of treatment in this context, providing a structured and effective approach for high-grade CNS lymphoma. Overall, this case underscores the critical importance of timely diagnosis, vigilant clinical monitoring, and individualized therapeutic strategies to optimize outcomes in patients with aggressive oncologic disorders.
